# Tumor-suppressor miRNA-27b-5p regulates the growth and metastatic behaviors of ovarian carcinoma cells by targeting CXCL1

**DOI:** 10.1186/s13048-020-00697-6

**Published:** 2020-08-11

**Authors:** Chun Hua Liu, Xue Ning Jing, Xiao Lan Liu, Shan Yong Qin, Min Wei Liu, Chun Hong Hou

**Affiliations:** 1 Obstetrics Department, Jiaozhou Central Hospital of Qingdao, Jiaozhou, Shandong China; 2 Shandong College of Traditional Chinese Medicine, Yantai, Shandong China; 3 School Hospital, Shandong Women’s University, Jinan, Shandong China; 4 Gynecology Ward, Heze Municipal Hospital, No. 2888 Caozhou Road, Heze, 274031 Shandong China

**Keywords:** Ovarian cancer, MiR-27b-5p, Migration, Invasion, CXCL1

## Abstract

**Background:**

MicroRNAs (miRNAs) play crucial functions in the progression of ovarian cancer. MicroRNA-27b-5p (miR-27b-5p) has been identified as a cancer-associated miRNA. Nevertheless, the expression profile of miR-27b-5p and its functions in ovarian cancer are unexplored.

**Methods:**

qRT-PCR and western blot analysis were used to detect the levels of miR-27b-5p and C-X-C motif chemokine ligand 1 (CXCL1). The impact of miR-27b-5p on ovarian cancer cells proliferation, migration and invasion in vitro were investigated using Cell Counting Kit-8 (CCK8), wound healing and Transwell, respectively. The expression of matrix metalloprotein-2/9 (MMP-2/9) were measured using immunofluorescence staining. Bioinformatics and luciferase reporter analysis were used to predict the target of miR-27b-5p. The growth of ovarian cancer cells in vivo was evaluated using transplanted tumor model.

**Results:**

Here, we demonstrated that miR-27b-5p was downregulated in ovarian carcinoma cells and clinical specimens. Higher expression of miR-27b-5p was associated with an unfavorable overall survival in patients with ovarian cancer. Upregulation of miR-27b-5p decreased the viability, migration ability and invasion capacity of SKOV3 and OVCAR3 cell. MiR-27b-5p also inhibited the growth of SKOV3 cell in nude mice. Additionally, we verified that CXCL1 was a target of miR-27b-5p in ovarian carcinoma cells. Restoring the expression of CXCL1 abolished the inhibitory impacts of miR-27b-5p in ovarian cancer carcinoma cells.

**Conclusion:**

This research revealed that miR-27b-5p restrained the progression of ovarian carcinoma possibly via targeting CXCL1.

## Introduction

Ovarian carcinoma is still one of the most lethal gynecological malignancies. Most deaths from ovarian cancer are attributed to distant metastasis. Metastasis, which is a multistep process, allows tumor cell diffuse from the primary sites to distant tissues [1, 2]. Increasing investigations have demonstrated that the loss of cancer suppressors and the upregulation of oncogenes are associated with cancer cell metastasis [3, 4]. Hence, investigations into the molecular mechanisms of cancer cell metastasis may assist in the exploration of targeted therapies to improve the clinical outcomes of patients with ovarian cancer.

MiRNAs are a class of non-coding RNAs and modulate the expressions of target genes via binding to the 3′-untraslated region (3′-UTR) of mRNAs [5]. Various miRNAs participate into the progression of malignant cancers [6–8]. For instance, deregulation of miR-194 contributes to colorectal carcinogenesis through targeting AKT Serine/Threonine Kinase 2 (AKT2) [9]. MiR-143-3p serves as a tumor suppressor via modulating the growth, invasion and epithelial-mesenchymal transition (EMT) by regulating KH Domain Containing RNA Binding-5 (QKI-5) in esophageal squamous cell carcinoma [10]. MiRNA-7-5p inhibits cell growth, induces cell cycle arrest and cell apoptosis via modulating Paired Box 2 (PAX2) in non-small cell lung cancer (NSCLC) [11]. In ovarian cancer, miRNA-141 enhances the anoikis resistance of metastatic ovarian carcinoma cell by regulating Kruppel Like Factor 12/Sp1 Transcription Factor (KLF12/Sp1) axis [12].

Recently, a report has identified that miR-27b-3p serve as a cancer suppressor in breast carcinoma stem cell generation through inactivating Ectonucleotide Pyrophosphatase/Phosphodiesterase 1 (ENPP1), to attenuate chemoresistance ability [13]. Furthermore, miR-27b-3p improves the sensitivity of liver and kidney cancer cell to anti-cancer drugs via activating p53-dependent apoptosis and reducing Cytochrome P450 Family 1 Subfamily B Member 1 (CYP1B1)-mediated drug detoxication [14]. In ovarian cancer, miRNA-27b acts as an inhibitor of ovarian carcinoma-mediated vasculogenic mimicry via repressing the expression of VE-cadherin [15]. Nevertheless, the underlying role of miR-27b-5p in ovarian cancer has no reports and needs further research.

In our study, we evaluated the possible roles of miR-27b-5p-CXCL1 axis in the aggressive ability of ovarian carcinoma cell. We verified that the expression of miR-27-5p was distinctly lower in ovarian carcinoma samples and its low level was associated with poor overall survival of patients with ovarian cancer. Furthermore, upregulation of miR-27b-5p restrained ovarian cancer cell growth, colony formation capacity and aggressive phenotypes in vitro, and tumorigenicity in vivo. In addition, CXCL1 was ascertained as a downstream target of miR-27b-5p in ovarian cancer and restoring CXCL1 expression counteracted the suppressive effects of miR-27b-5p. Altogether, miR-27b-5p repressed the progression of ovarian carcinoma through regulating CXCL1.

## Materials and methods

### Ovarian cancer tissues

Total 45 pairs of ovarian carcinoma tissues and adjacent samples (located > 3 cm from cancer tissue) were obtained from patients who received surgical resection in Jinan Central Hospital. Patients did not receive chemotherapy or radiotherapy before surgery. All samples were snap-frozen in liquid nitrogen and storage at − 80 °C. This research was approved by the Ethics Committee of Jinan Central Hospital. Informed consent was obtained before this research.

### Cell transfection

Ovarian cancer cells (SKOV3, Caov-3, A2780 and OVCAR3) and human ovarian surface epithelial cell line, HOSEpiC were obtained from Nanjing KeyGen Biotech (Nanjing, Jiangsu, China). Cell lines were maintained in RPMI-1640 medium supplement with 10% FBS (Thermo Fisher Scientific), 100 μg/ml streptomycin and 100 μg/ml penicillin in 5% CO_2_ at 37 °C. MiR-27b-5p mimics or miRNA negative control (NC) mimics were synthesized by GenePharma (Shanghai, china). MiR-27b-5p stable transfected cells were constructed by using over-expressing miR-27b-5p lentivirus carrier (Vigenebio, Shandong, China). CXCL1 cDNA sequences were cloned into a pCDNA3.1 vector (Thermo Fisher Scientific). miR-27b-5p mimics combination with pCDNA3.1 vector carrying CXCL1 were transfected into SKOV3 or OVCAR3 cell using Lipofectamine 3000 (Thermo Fisher Scientific).

### Cell proliferation

Cells (2 × 10^3^) were cultured into 96-well plates and incubated for 1 day, 2 days, 3 days, 4 days or 5 days. Then, cell counting kit-8 (CCK-8) solution (Beyotime Biotechnology, Nanjing, China) was added into 96-well plates. After 2 h, the OD in each well was determined at 450 nm.

### Colony formation assay

1 × 10^3^ cells was cultured in six-well plates and cultured for total two weeks. Then, the colonies in plate were fixed by 4% formaldehyde and dyed with 1% crystal violet. The number of cell colonies in plate was counted by using a microscope.

### Migration assay

Cells were culture in six-well plates. When the cells reached about 80–90% confluence, wounds were made using a sterile 100 μl pipette tip. The scratch area was imaged at a certain time interval (0 h and 24 h) and cell migration capacity was calculated using formula as follow: Migration (%) = (0 h width of scratch - 24 h width of scratch)/0 h width of scratch × 100%.

### Invasion assay

2 × 10^5^ cells were seeded into the upper chamber of a Matrigel-coated Transwell chamber in serum-free medium. The lower chamber was supplemented with medium containing 20% FBS as a chemoattractant. The cells were incubated for 48 h, and the chamber was fixed with 10% neutral formalin for more than 4 h. The cells were stained with 1% crystal violet (Beyotime). Then the number of invaded cells was counted under a microscope (Olympus).

### Soft agar colony assay

1 × 10^4^ SKOV3 or OVCAR3 cells were cultured in 0.35% agar in the middle of the agar. In 24 well plates, 0.5% agar was added into the bottom and 0.35% agar was added into the top. Cells in plates were maintained for two weeks. Finally, colonies were photographed and counted.

### Immunoblotting

Total proteins were harvested using RIPA (Beyotime Biotechnology). Proteins were separated using 8% SDS-PAGE and the separated proteins were transferred onto PVDF membranes (Millipore, Braunschweig, Germany). PVDF membrane was incubated with CXCL1 or GAPDH (Abcam, Cambridge, UK) at 4 °C for overnight. After that, the membranes were incubated with a secondary antibody (Beyotime Biotechnology). Finally, bands were measured by using enhanced chemiluminescence (ECL) detection system.

### qRT-PCR assay

RNAs were abstracted by using TRIzol and First-strand cDNA was constructed with 1 μg RNA by using a Reverse Transcription kit (Takara Bio, Dalian, China). The qRT-PCR was conducted on 7500 Real-Time PCR system using a SYBR Green One kit (Takara Bio). U6 and GAPDH were served as endogenous controls. The primers were as following (sense and antisense): U6: GGAACGATACAGAGAAGATTAGC and TGGAACGCTTCACGAATTTGCG; miR-27b-5p: CAAAUUCGGAUCUACAGGGUAUU and UACCCUGUAGAUCCGAAUUUGUG; CXCL1: AACCGAAGTCATAGCCACAC and GTTGGATTTGTCACTGTTCAGC; GAPDH: AAAGGTGGAGGAGTGGGT and GGGAAACTGTGGCGTGAT. The data were calculated using 2^-∆∆Ct^ method.

### Luciferase reporter gene analysis

The fragment of CXCL1 3′-UTR with wild-type miR-27b-5p binding sites (wt) or mutated binding sites (mut) was inserted into psiCHECK-2 vector (Promega, Madison, WI, USA). The vector combination with miR-27b-5p was transfected into SKOV3 or OVCAR3 cells. Luciferase activities in SKOV3 and OVCAR3 cells were assessed by using Luciferase assay kit (Promega).

### Transplanted tumor model

The stable over-expressed miR-27b-5p SKOV3 cells (5 × 10^6^) or control group were subcutaneously injected into BALB/C nude mice (*n* = 3 in each group). Tumor volumes were measured and calculated. Tumor volume = 0.5 × length×width^2^. After five weeks, mice were sacrificed. The tumor tissues were fixed and subjected for hematoxylin and eosin (H&E) staining and immunohistochemical (IHC) assay using CXCL1 antibody. Animal experiment was approved by the Animal Care Committee and Use Committee of Jinan Central Hospital.

### Statistical analysis

All statistical analyses were conducted using Graphpad prism. Results were presented as mean ± SD. The differences were analyzed by unpaired Student’s *t*-test or one-way ANOVA followed by Tukey’s *post-hoc* test. The differences of miR-26b-5p and CXCL1 levels in ovarian carcinoma and adjacent normal tissues were using paired *t*-test. The relationship between the miR-27b-5p and CXCL1 was detected using Pearson’s correlation analysis. Survival estimation was analyzed using Kaplan-Meier method. *P* value less than 0.05 was statistically significant.

## Results

### MiR-27b-5p is downregulated in ovarian carcinoma

**Fig. 1:**
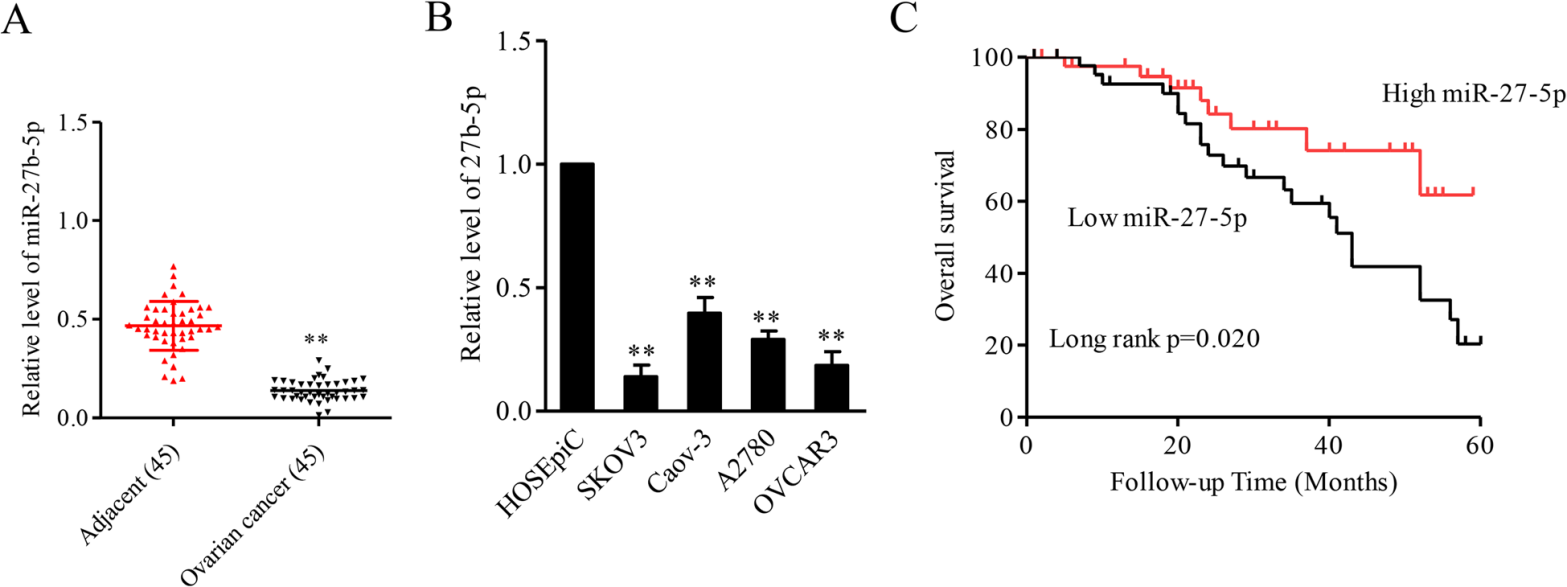
MiR-27b-5p is downregulated in ovarian cancer. **A.** qRT-PCR for determining relative levels of miR-27b-5p in ovarian cancer and adjacent tissues (*n* = 45). ^**^P < 0.05 vs. adjacent. **B.** qRT-PCR detection of miR-27b-5p expression in ovarian carcinoma cells and human ovarian surface epithelial cell line, HOSEpiC. ^**^P < 0.05 vs. HOSEpiC. **C.** Kaplan-Meier analysis of the relationship between miR-27b-5p level and ovarian cancer patients’ overall survival

Firstly, we checked the expressions of miR-27b-5p in human ovarian carcinoma samples. The result of qRT-PCR analysis using 45 cases of ovarian carcinoma and adjacent tissues suggested that miR-27b-5p was frequently downregulated in ovarian carcinoma compared with in adjacent tissues (Fig. 1A). Results also indicated that miR-27b-5p was drastically downregulated in ovarian carcinoma cells compared with in human ovarian surface epithelial cell line, HOSEpiC (Fig. 1B). The relationship between the dysregulation of miR-27b-5p and the clinicopathological features of patients with ovarian cancer was future analyzed. As showed in Supplementary Table 1, the low level of miR-27b-5p was connected to the advance tumor stage and more metastasis of ovarian cancer. Finally, survival analyses revealed that the decreased expression of miR-27b-5p was related to a poor overall survival of patients with ovarian cancer (Fig. 1C). These findings indicate that miR-27b-5p exerts key roles in the tumorigenesis of ovarian cancer.

### MiR-27b-5p inhibits ovarian carcinoma cell growth and colony formation

**Fig. 2:**
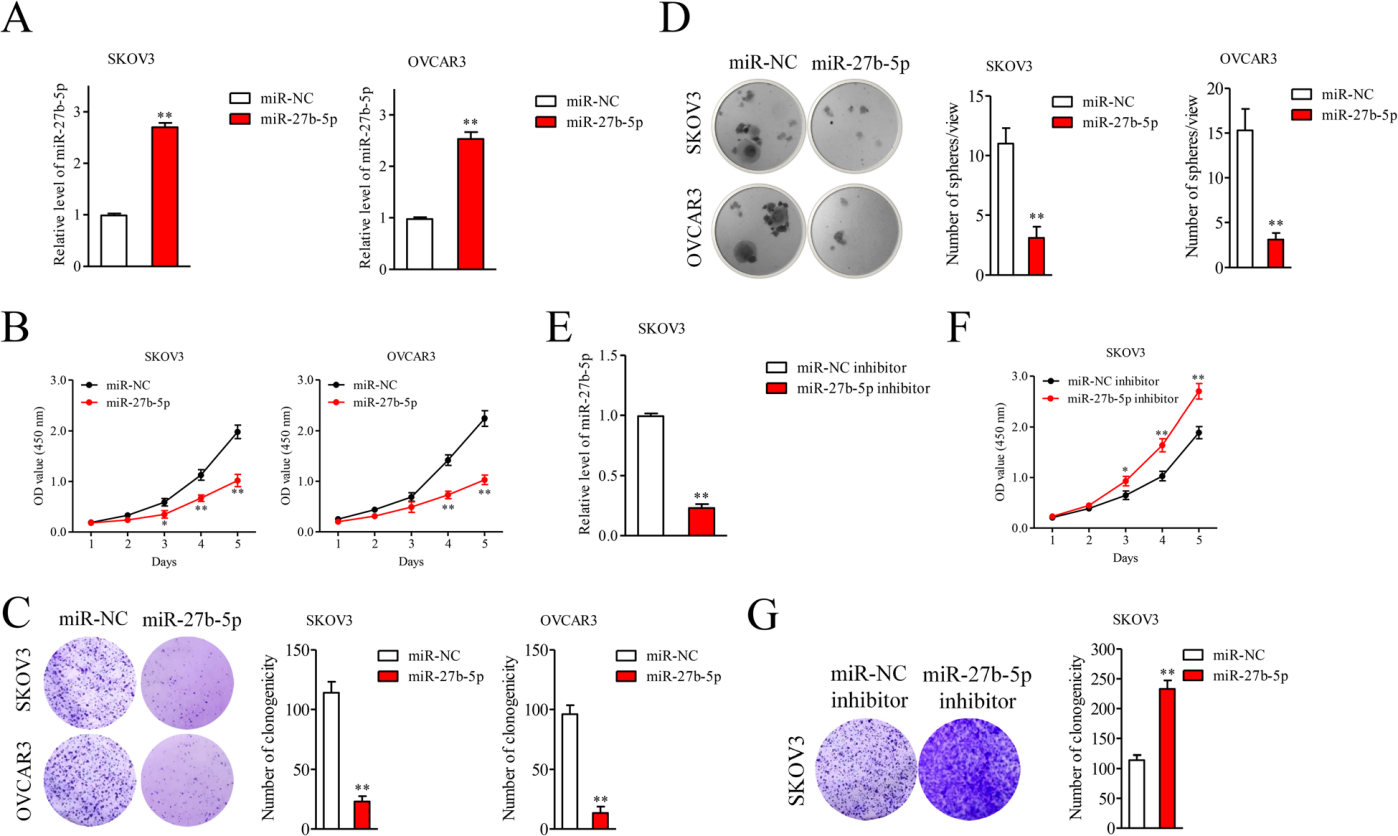
MiR-27b-5p inhibits the proliferation of ovarian cancer cells in vitro. **A.** SKOV3 and OVCAR3 cells were transfected with miR-NC or miR-27b-5p mimics and the level of miR-27b-5p was analyzed by qRT-PCR assay. **B.** CCK-8 assay of SKOV3 and OVCAR3 transfected with miR-NC or miR-27b-5p. **C.** Colony formation assay was conducted to evaluated cell proliferative ability in miR-27b-5p overexpressing SKOV3 and OVCAR3 cells. **D.** Soft agar colony formation assay was conducted to evaluated cell proliferative ability in miR-27b-5p overexpressing SKOV3 and OVCAR3 cells. ^**^P < 0.05 vs. miR-NC. **E.** SKOV3 cells were transfected with miR-NC inhibitor or miR-27b-5p inhibitor and the level of miR-27b-5p was analyzed by qRT-PCR assay. **F.** CCK-8 assay of SKOV3 cells transfected with miR-NC inhibitor or miR-27b-5p inhibitor. **G.** Colony formation assay was carried out using SKOV3 transfected with miR-NC inhibitor or miR-27b-5p inhibitor. ^**^P < 0.05 vs. miR-NC inhibitor

To better illuminate the role of miR-27b-5p, SKOV3 and OVCAR3 cells were transfected with miR-27b-5p mimics. The qRT-PCR analysis shown that miR-27b-5p transfection raised miR-27b-5p levels in both SKOV3 and OVCAR3 cells (Fig. 2A). Results from CCK-8 assays indicated that introduction of miR-27b-5p remarkably reduced the cell viabilities of A2780 and OVCAR3 cells (Figs. 2B). Moreover, miR-27b-5p distinctly suppressed the colony formation abilities of SKOV3 and OVCAR3 cells in vitro (Fig. 2C). Consistently, transfection of miR-27b-5p suppressed colony formation of ovarian carcinoma cells in soft agar colony assay (Fig. 2D). Meanwhile, SKOV3 cells were treated with miR-27b-5p inhibitor to degrade the level of miR-27b-5p. As shown in Fig. 2E, miR-27b-5p inhibitor remarkably degraded the level of miR-27b-5p. As expected, miR-27b-5p silencing increased the cell viability and colony formation ability of SKOV3 cells in vitro (Fig. 2F-2G). These observations suggest that miR-27b-5p suppresses SKOV3 and A2780 cells growth in vitro.

### MiR-27b-5p modulates ovarian cancer cell migration and invasion

**Fig. 3:**
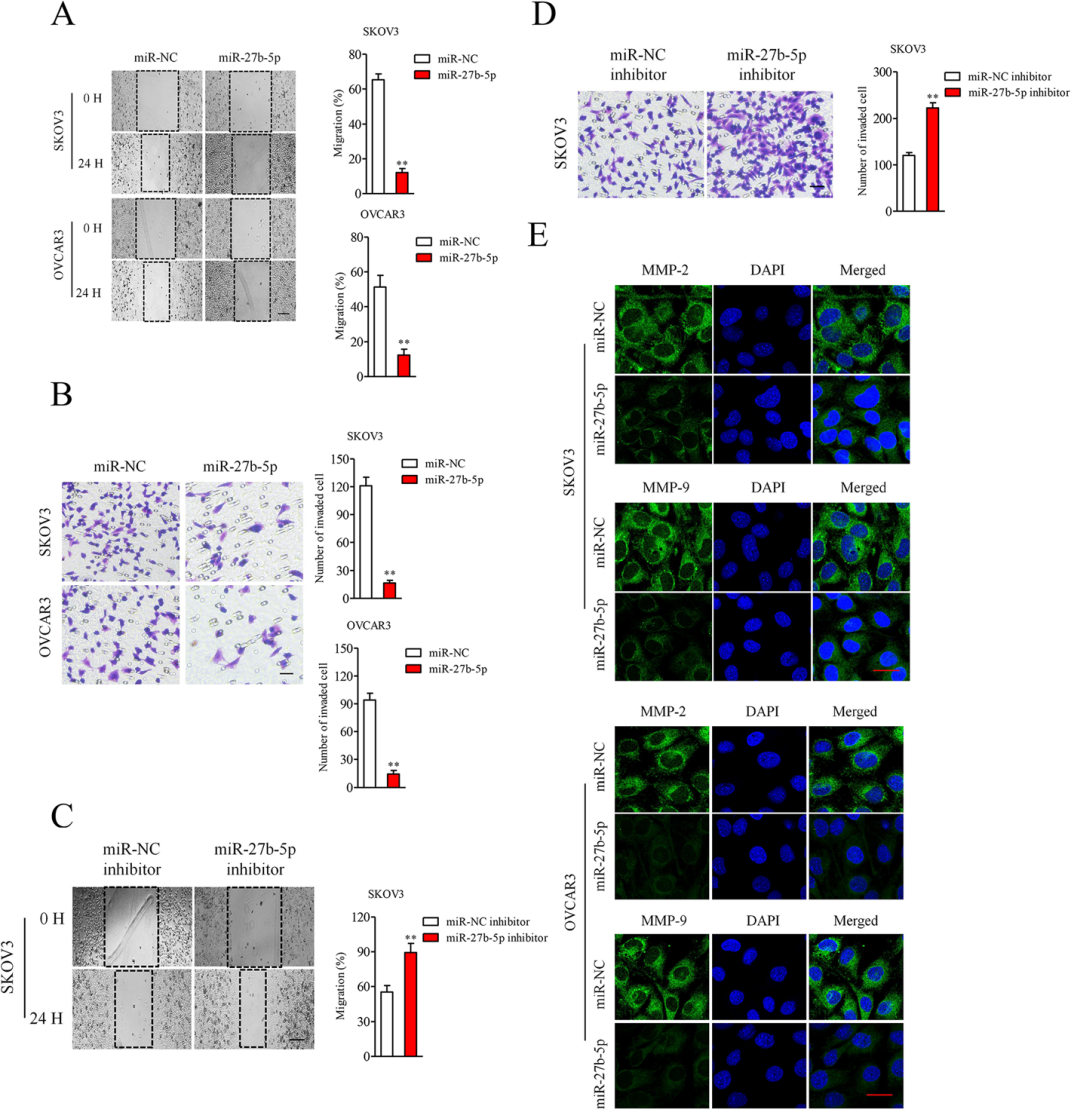
MiR-27b-5p inhibits ovarian cancer cell migration and invasion. **A.** Wound heal assay was carried out to evaluate the migration in miR-NC and miR-27b-5p mimics transfected SKOV3 and OVCAR3 cells. **B.** Transwell assay was carried out to evaluate the invasion in miR-NC and miR-27b-5p mimics transfected SKOV3 and OVCAR3 cells. ^**^P < 0.05 vs. miR-NC. **C.** Wound heal assay shown the migration in miR-NC inhibitor and miR-27b-5p inhibitor transfected SKOV3 cells. **D.** Transwell assay shown the invasion in miR-NC inhibitor and miR-27b-5p inhibitor transfected SKOV3 cells. ^**^P < 0.05 vs. miR-NC inhibitor. **E.** Immunofluorescence analysis of MMP-2/9 in SKOV3 and OVCAR3 cells transfected with miR-NC or miR-27b-5p

We then conducted wound closure and Transwell invasion assay to analyze the action of miR-27b-5p in SKOV3 and OVCAR3 cells mobility and invasiveness. We observed that transfection of miR-27b-5p markedly reduced the migrate and invasion abilities of SKOV3 and OVCAR3 cells (Fig. 3A-3B). Nevertheless, downregulation of miR-27b-5p significantly impaired the migration and invasion abilities of SKOV3 cells (Fig. 3C-3D). Interestingly, the expression levels of MMP-2 and MMP-9 were decreased in miR-27b-5p overexpressing SKOV3 and OVCAR3 cells as demonstrated by immunofluorescence staining assay (Fig. 3E). These data demonstrate that miR-27b-5p is a regulator of ovarian cancer cell mobility and invasion.

### MiR-27b-5p targets CXCL1 in ovarian cancer cell

**Fig. 4:**
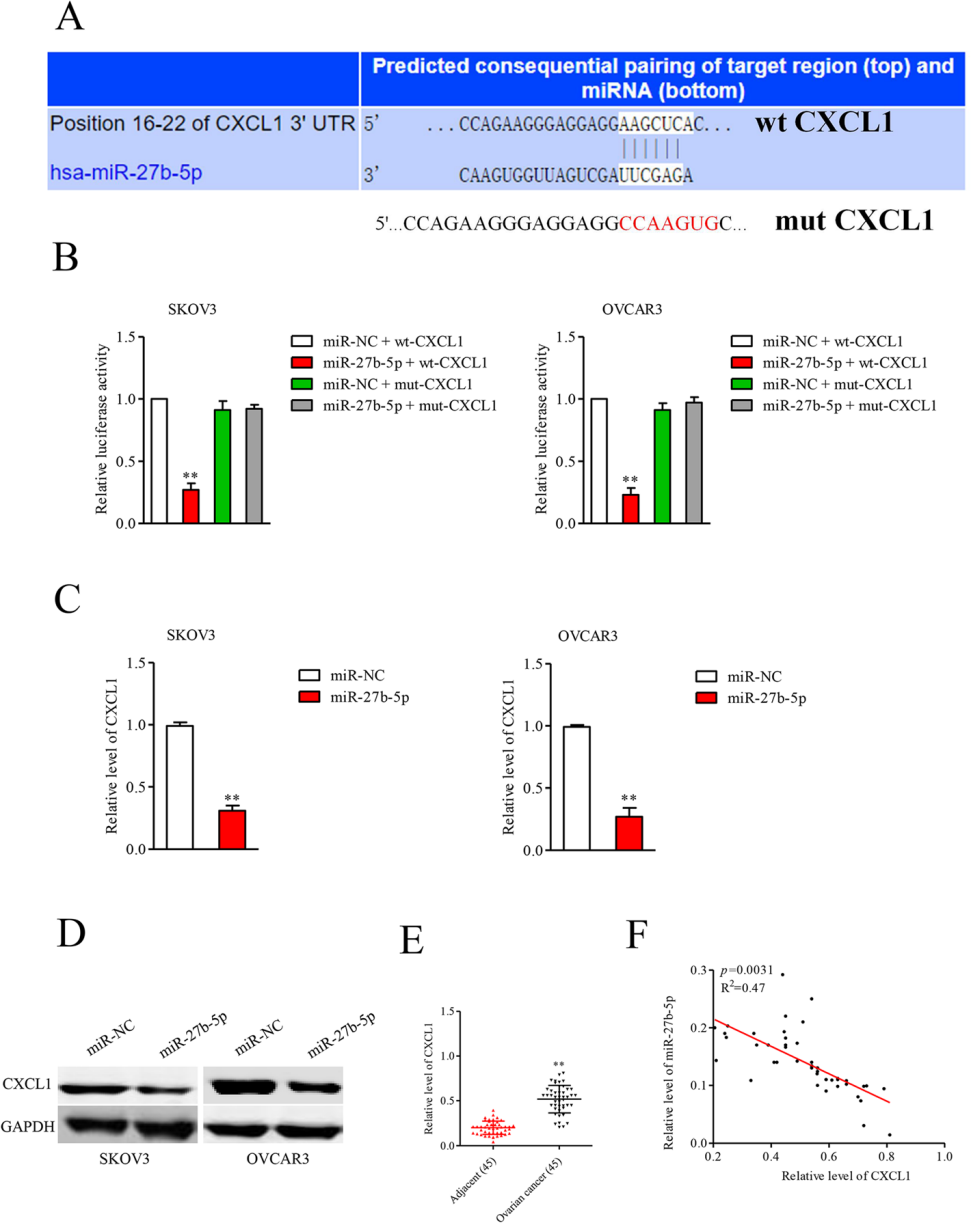
Identification of CXCL1 as a target of miR-27b-5p. **A.** miR-27b-5p and its wild-type (wt) binding sites in the 3′-UTR of CXCL1. The mutant binding sites (mut) were produced in the complementary site for the seed region of miR-27b-5p. **B.** psiCHECK-2 carrying CXCL1–3′-UTR wt or psiCHECK-2 carrying CXCL1–3′-UTR mut, along with miR-27b-5p mimics or miR-NC, were cotransfected into SKOV3 and OVCAR3 cells. The luciferase activity was detected using a luciferase reporter assay system. ^**^P < 0.05 vs. miR-NC. **C.** The mRNA levels of CXCL1 in miR-27b-5p overexpressing SKOV3 and OVCAR3 cells were examined using qRT-PCR. **D.** The protein levels of CXCL1 in miR-27b-5p overexpressing SKOV3 and OVCAR3 cells were examined using western blot analysis. **E.** The level of CXCL1 in ovarian cancer tissues and adjacent tissues were assessed using qRT-PCR. ^**^P < 0.01 vs. adjacent tissues. **F.** Spearman’s correlation analysis was utilized to examine the expression correlation between miR-27b-5p and CXCL1 mRNA in ovarian cancer tissues

Bioinformatics analysis tool (http://www.targetscan.org/vert_71/) was selected to find the target gene of miR-27b-5p. Among these candidates, CXCL1 (Fig. 4) was predicted as a downstream gene of miR-27b-5p, owing to CXCL1 has been implicated in the malignant phenotypes of ovarian cancer [16, 17]. Then, the result of luciferase reporter experiments implied that transfection of miR-27b-5p impaired the luciferase activities in A2780 and OVCAR3 cells transfected with reporter plasmid containing wt-CXCL1. Notably, the luciferase activities in cells transfected with reporter plasmid containing mut-CXCL1 were not inhibited by miR-27b-5p, which indicating that miR-27b-5p directly bound to the 3′-UTR of CXCL1 (Fig. 4B). In addition, the results of qRT-PCR and immunoblotting analysis suggested that the mRNA (Fig. 4C) and protein (Fig. 4D) levels of CXCL1 were significantly reduced in miR-27b-5p overexpressing A2780 and OVCAR3 cells. To elucidate the correlation between miR-27b-5p and CXCL1, the level of CXCL1 in ovarian carcinoma tissue was measured, and we found that the mRNA level of CXCL1 was upregulated in ovarian carcinoma tissue when compared to in adjacent sample (Fig. 4E). Finally, an inversely correlation between miR-27b-5p and CXCL1 in ovarian cancer tissue was confirmed through Spearman’s correlation analysis (Fig. 4F). Altogether, these findings imply that miR-27b-5p regulates the level of CXCL1 in ovarian cancer cell.

### Restoring CXCL1 expression counteracts the suppressive effects of miR-27b-5p

**Fig. 5:**
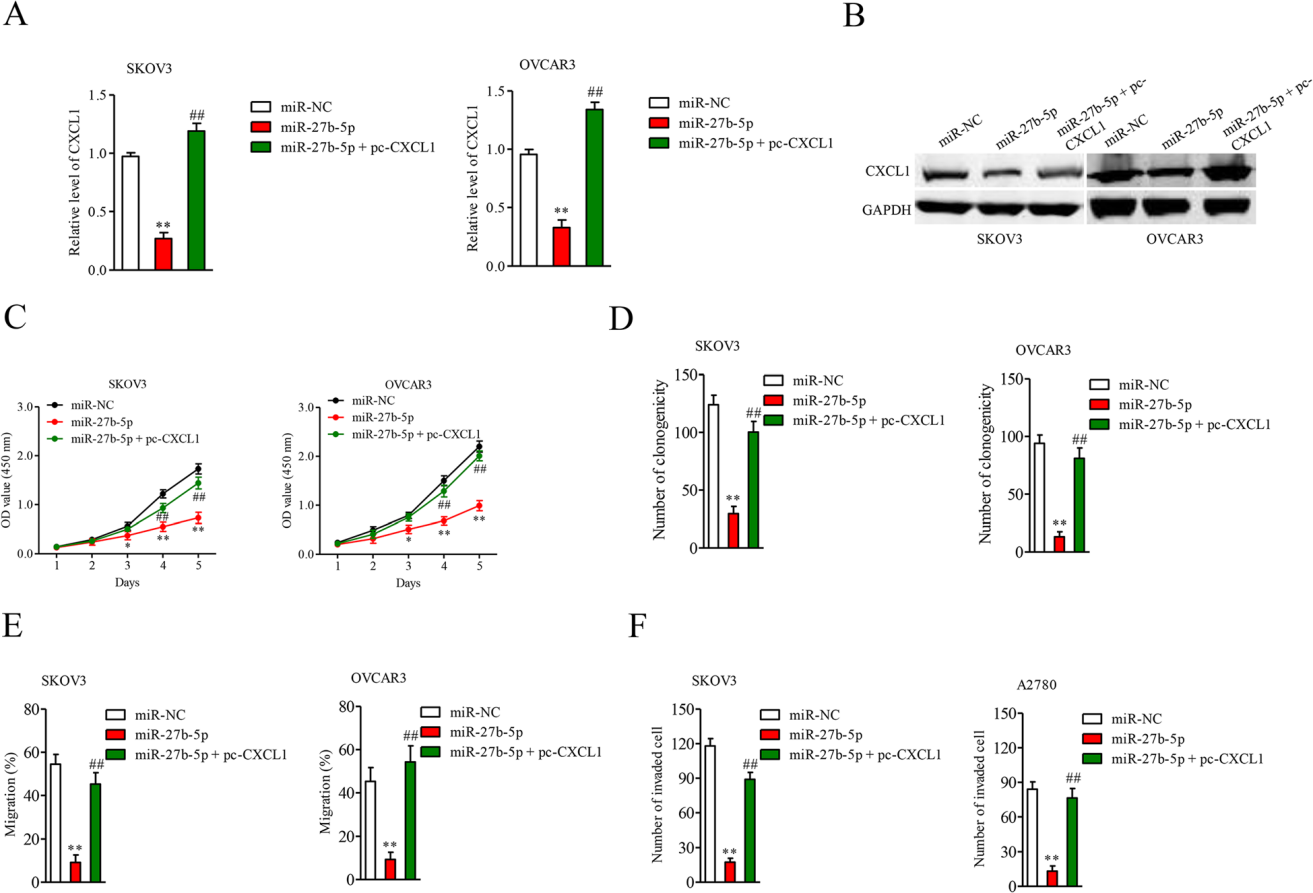
Overexpression of CXCL1 reverses the effects of miR-27-5p. **A.** SKOV3 and OVCAR3 cells were co-transfected with miR-27b-5p mimics and pc-CXCL1 or pcDNA3.1. The level of CXCL1 was detected by qRT-PCR. **B.** Western blot analysis was utilized for the detection of CXCL1 protein expression in SKOV3 and OVCAR3 cells following pcDNA3.1 or pc-CXCL1 transfection. **C.** SKOV3 and OVCAR3 cells were co-transfected with miR-27b-5p mimics and pc-CXCL1 or pcDNA3.1. Following transfection, the proliferations of SKOV3 and OVCAR3 cells were examined using CCK-8 assays **D.** The colony formation of SKOV3 and OVCAR3 cells were examined. **E.** The migration of SKOV3 and A2780 cells were examined using wound healing assay. **F.** The invasion of SKOV3 and OVCAR3 cells were examined using Transwell invasion assay. ^**^P < 0.05 vs. miR-NC, ^##^*P* < 0.05 vs. miR-27b-5p + pc-CXCL1

Then, several rescue experiments were carried out to ensure that CXCL1 is essential for the functions of miR-27b-5p in ovarian cancer. Firstly, pcDNA3.1 carrying CXCL1 (pc-CXCL1) or control pcDNA3.1 vector was transfected into A2780 and OVCAR3 cells. Immunoblotting analysis and qRT-PCR assay verified that transfection of pc-CXCL1 rescued the expression of CXCL1 in ovarian cancer cell in the presence miR-27b-5p (Fig. 5A-5B). Upregulation of miR-27b-5p inhibited SKOV3 and OVCAR3 cells viability (Fig. 5C) and colony formation ability (Fig. 5D) in vitro, whereas reintroduction of CXCL1 abolished these effects. Meanwhile, miR-27b-5p hindered the migration and invasion of SKOV3 and OVCAR3 cells, whereas reintroduction of CXCL1 abrogated these effects (Fig. 5E-5F). Altogether, miR-27b-5p exerts suppressive roles in ovarian cancer progression through decreasing CXCL1 expression.

### MiR-27b-5p inhibits ovarian cancer cell growth in mice

**Fig. 6:**
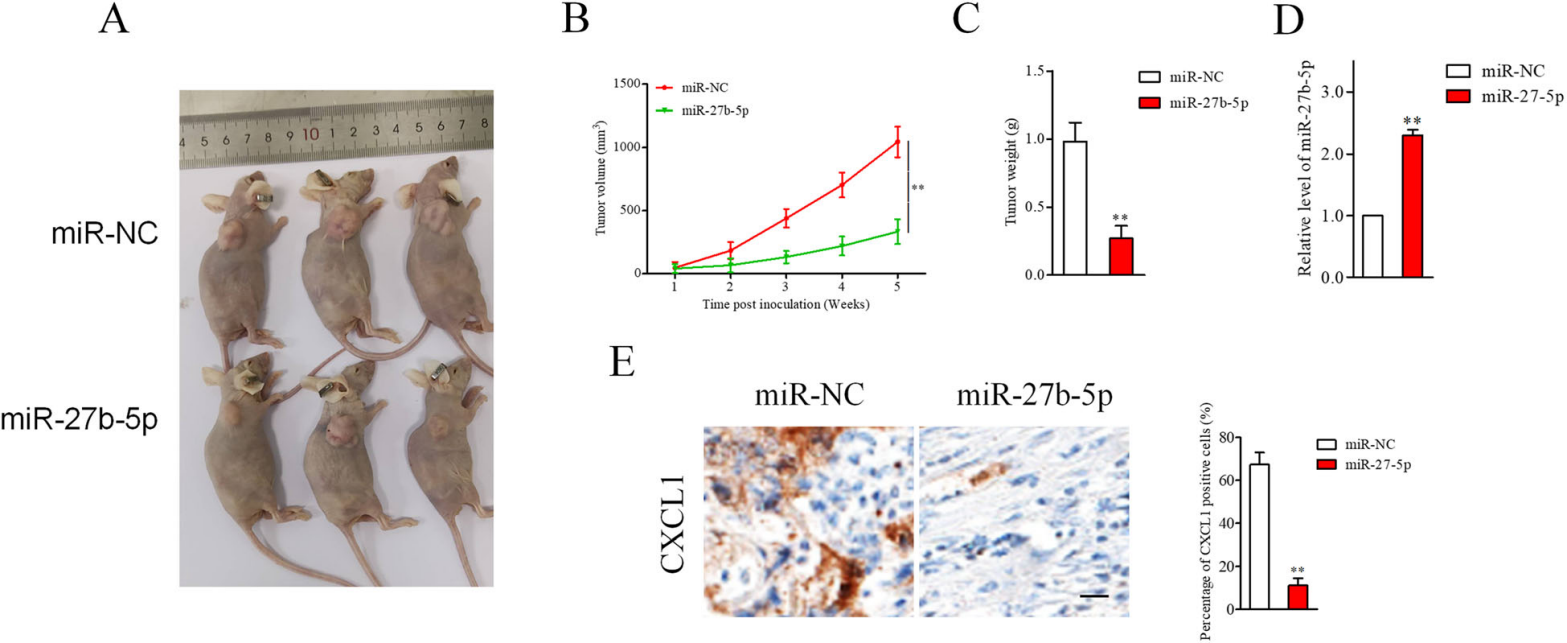
miR-27b-5p inhibits tumor growth of SKOV3 cell in vivo. **A.** miR-NC or miR-27b-5p stable transfected SKOV3 cells were injected into nude mice. Mice were sacrificed at 35 days post-injection. **B.** Overexpressed miR-27b-5p suppressed tumor growth. **C.** Analysis of tumor weight after xenograft tumor removed. **D**. The level of miR-27b-5p in tumor tissue was detected using qRT-PCR assay. **E.** IHC staining for CXCL1 in xenograft tumor and CXCL1 positive staining was shown using histogram. ^**^P < 0.05 vs. miR-NC

To assess whether overexpression of miR-27b-5p affects ovarian cancer cell progression in vivo, nude mice was subcutaneously inoculated with miR-27b-5p stable transfected SKOV3 cells. Tumor volume in each group was detected each week, and nude mice were sacrificed after five weeks. As shown in Fig. 6A-6B, the tumor volume of tumor tissue formed by miR-27b-5p stably transfected SKOV3 cells was markedly smaller than tumor tissue derived from miR-NC transfected cells. Consistently, the tumor weight in mice inoculated with miR-27b-5p stably transfected SKOV3 cell was markedly smaller than tumors in the miR-NC group (Fig. 6C). qRT-PCR assay revealed that the level of miR-27b-5p was markedly higher in miR-27b-5p group than that in miR-NC group (Fig. 6D). More importantly, IHC staining confirmed that the expression of CXCL1 was significantly inhibited in miR-27b-5p transfected group (Fig. 6E). Therefore, miR-27b-5p exerted cancer inhibiting property in SKOV3 cell growth in vivo.

## Discussion

The dysregulations of miRNAs have been proved as crucial drivers in ovarian cancer metastasis and development. But, the level of miR-27b-5p is yet unknown in human ovarian carcinoma. In the study, we observed that miR-27b-5p was downregulated in clinical ovarian carcinoma tissue. Moreover, miR-27b-5p was lowly expressed in ovarian cancer cells compared with in human ovarian surface epithelial cell line, HOSEpiC. We also found that miR-27b-5p low level was connected to the advanced stage and metastasis in ovarian cancer. The survival analyses suggested that patients who had low level of miR-27b-5p exhibited worse overall survival. All these observations indicated that miR-27b-5p exerted a critical action in the progression of ovarian cancer.

Increasing researches have demonstrated that miRNAs exert vital roles in various biological process associated with cancer, including cell proliferation, apoptosis, metastasis and chemotherapy-resistant [18–21]. Substantive investigations reveal that the aberrant levels of miRNAs are strongly linked to the growth and metastasis of ovarian cancer. For instance, miR-138 represses ovarian carcinoma cell metastasis via modulating the expressions of SRY-Box Transcription Factor 4 (SOX4) and Hypoxia Inducible Factor 1 Subunit Alpha (HIF-1α) [22]. MiRNA-375 represses the growth, drug sensitivity and metastasis of ovarian carcinoma cell by targeting Paired Box 2 (PAX2) [23]. Previous investigation also reports that miR-27b suppresses NSCLC cell growth and invasion through regulating LIM Domain Kinase 1 (LIMK1) [24]. MiR-27b represses the growth and progression in neuroblastoma cell by targeting Peroxisome Proliferator Activated Receptor Gamma (PPARγ) [25]. In our study, we revealed that transfection of miR-27b-5p inhibited ovarian cancer cell viability and colony formation in vitro. Meanwhile, upregulation of miR-27b-5p remarkably reduced the migration ability and invasiveness of ovarian carcinoma cell.

The classic pattern of miRNAs-regulating their target genes are to bind with the 3′-UTR of genes. The 3′-UTR prediction tool (http://www.targetscan.org/vert_71/) displayed that the binding between miR-27b-5p and CXCL1 3′-UTR regions was identified. The result of luciferase reporter gene analysis proved that miR-27b-5p bound to the 3′-UTR of CXCL1. Furthermore, the expression of CXCL1 was significantly impaired by miR-27b-5p in SKOV3 and OVCAR3 cell. Previous report has indicated that CXCL1 induces the proliferation of ovarian carcinoma cell through transactivation of epidermal growth factor receptor (EGFR) [16]. Serum CXCL1 is a novel circulating tumor marker for the differential diagnosis between benign ovarian masses and ovarian cancer [26]. In the current study, restoring CXCL1 expression counteracted the suppressive effects of miR-27b-5p in ovarian cancer cell. Finally, miR-27b-5p inhibited ovarian carcinoma cell growth in vivo and decreased the expression of CXCL1 in tumor tissue.

Our study revealed that miR-27b-5p was down-expressed in ovarian carcinoma and its low level was connected to the advance stage and unfavorable prognosis of patients. Mechanistically, miR-27b-5p repressed the tumor growth and metastatic behaviors of ovarian cancer cell possibly via suppressing CXCL1. Our observations might bring new insights into ovarian cancer progression and revealed a novel mechanism by which miR-27b-5p regulated the proliferation and malignant metastatic phenotypes of ovarian carcinoma cell via targeting CXCL1.

## Supplementary information

**Additional file 1 Supplementary Table 1.** Relationship between the expression of miR-27b-5p and clinicopathological parameters in patients with ovarian cancer.

## Data Availability

All data generated or analysed during this study are included in this published article.
