# Supplementary material for: Tumor-suppressor miRNA-27b-5p regulates the growth and metastatic behaviors of ovarian carcinoma cells by targeting CXCL1
Source: J Ovarian Res. 2020 Aug 11;13:92. doi: 10.1186/s13048-020-00697-6 (PMC7418439; doi:10.1186/s13048-020-00697-6)
Supplement: Supplementary file 1 — Additional file 1 Supplementary Table 1. Relationship between the expression of miR-27b-5p and clinicopathological parameters in patients with ovarian cancer. [file 13048_2020_697_MOESM1_ESM.docx]

**Supplementary Table 1.** Relationship between the expression of miR-27b-5p and clinicopathological parameters in patients with ovarian cancer.

| **Parameter** | **miR-27-5p** | | ***p*-value** |
| --- | --- | --- | --- |
|  | **High (n)** | **Low (n)** |  |
| **Age (years)** |  |  | 0.224 |
| <50 | 6 | 8 |  |
| ≥50 | 14 | 17 |  |
| **FIGO stage** |  |  | 0.036 |
| Early (I-II) | 8 | 4 |  |
| Advanced (III-IV) | 12 | 21 |  |
| **Lymph node metastasis** |  |  | 0.032 |
| Yes | 9 | 19 |  |
| No | 11 | 6 |  |

FIGO, international federation of gynecology and obstetrics according to the 2009 FIGO surgical staging system.
